# Supplementary material for: Evaluation of metronomic chemotherapy response using diffusion and dynamic contrast-enhanced MRI
Source: PLoS One. 2020 Nov 25;15(11):e0241916. doi: 10.1371/journal.pone.0241916 (PMC7688103; doi:10.1371/journal.pone.0241916)
Supplement: Attachment — Submitted filename: Response to Reviewers.doc [file pone.0241916.s001.doc]

The authors thank the reviewers and the editor for their constructive comments and insightful suggestions. We have revised the manuscript based on reviewer’s specific comments, as detailed in the point-by-point responses below. The changes are marked in the revised manuscript

**Reviewer 1.**

***R1. C1.*** *In the introduction the suppression of the mobilisation of CEPS is mentioned but not assessed, this would be an interesting addition to the manuscript to back up the vascular MRI data.*

**Response: *We agree with the reviewer that the information about CEPS would be aa good addition to the imaging data shown in this manuscript. Unfortunately, that was not included in this study. This was mentioned as a limitation of the study in the revised manuscript.***

***R1****.****C2****. What dose of Gd-DTPA is given? Please add concentration of solution given.*

**Response: *It was our oversight to use a wrong unit for the injection dose. We used 0.1 mmol/Kg body weight which is a commonly used dose of Gd-based contrast agent for DCE-MRI studies.***

***R1.C3****. The formulae for the EMM are not the same as in the referenced articles, please clarify these differences.*

**Response: *Thanks for the suggestion. We did not include the last exponential term of the EMM which is for the initial washout rate (gamma). This was following the observation in a previous study (Fan et al 2007) that the EMM with gamma = 0 was found to be adequate to describe benign and malignant lesion curves. We clarified this in the revised manuscript.***

***R1.C4.*** *A separate statistics section in the methods would be advantageous. Were the data tested for normality prior to the use of t tests? Were corrections applied to the t tests to mitigate for the multiple statistical comparisons made?*

**Response:** ***A separate statistical analysis section is now added in the revised manuscript, following the suggestion. Please note that our sample size is too small to do a normality test. We clarified in the revised manuscript that the p-values reported in this study are without a correction for multiple comparisons.***

***R1.C5*** *Please add additional information to the figure legends so that they can be fully appreciated in isolation from the main text.*

**Response: *The legends were updated to add more information.***

***R1.C6.*** *In Figure 1 what are the 3 lines on the density/MRI maps? I would have expected 2 showing the ‘rim’ region but what is the third?*

**Response: *Thanks for the question. We used two blue lines to show a 0.2 mm-wide band immediately inside of the boundary of the tumor for the rim ROI, and then a red line to indicate the tumor core ROI. This has been clarified in the legend of Figure 1 in the revised manuscript.***

***R1.C7.*** *In the tumor volume section of the results, I think the author should be referring to tumor growth rather than cellular growth, also a reduction in tumor volume does not necessarily indicate a reduction in cell division, there could be an increase in cell death, this is not mentioned.*

**Response: *We agree with the reviewer. The first of paragraph of the result section regarding the tumor volume has been revised to refer to tumor growth rather than cellular growth.***

***R1.C8.*** *In figure 3 it is difficult to assess the enhancement patterns in the DCE images, it would be clearer if a pre-GdDTPA image was shown, or a representation of signal change. ROIs would also improve clarity.*

**Response: *Thank you for the suggestion. As the reviewer pointed out, contrast enhancements in 4T1 tumors are not quite clear, particularly in the core area. We followed the reviewer’s suggestion to add pre-Gd images and ROIs for the tumors in the figure, which is now updated as Figure 4.***

***R1.C9.*** *The first sentence in the ‘treatment response in DCE-MRI’ section of the results is misplaced, it should be in the introduction or discussion. I think the authors are also referring to vascular normalization, so the term standardized is not correct.*

**Response**: ***We agree with the reviewer on this point.*** ***The first two sentences in that section have been moved to discussion.***

***R1.C10.*** *An antiangiogenic effect would ordinarily be identified as a reduction in signal enhancement following Gd-DTPA administration but the authors are describing no change in contrast enhancement as ‘confiming the anti-angiogenic effect of 5FU’ but a reduction in enhancement in the control tumors as no response, please clarify these conclusions. The statement that the vascular changes ‘allow for Gd-DTPA to circulate through the tumor’ is inconsistent with the observation that enhancement is only seen in the rim of the tumors, and indeed the following sentence stating that chemotherapy cuts off the blood supply.*

**Response***:* ***We agree with the reviewer that it is typically expected that an antiangiogenic effect is observed as reduction in contrast enhancement. However, it has also been reported that such effect could be due to “over-pruning” of the tumor vasculature. On the other hand, a lower dose of antiangiogenic therapy could achieve vascular normalization where tumor perfusion can be increased or maintained for better drug delivery. We believe this is what we observe in this study with a relatively mild anti-angiogenic effect induced by MCT. These sentences regarding our interpretation of the data have been moved to discussion for further clarification in the revised manuscript.***

***R1.C11.*** *Please clarify how smaller q measurements relate to quicker contrast agent washout in figure 5, would showing beta values show this more clearly? The statement ‘MCT tumors had significantly more enhancements..’ is clumsy, do you refer to higher enhancement, more Gd-DTPA extravasation?*

**Response**: ***In this revision (now Figure 6), we added another plot to show beta values. There was no significant difference in beta values between the MCT and control groups, while there was a significant difference in q between the post-treatment data of the MCT and control groups. Thank you for pointing out our oversight about the description of the enhancement level. We changed it to higher enhancement.***

***R1.C12.*** *A higher ADC value cannot simply be attributed to more water being able to pass though cell membranes, cell death is often a major contributor to ADC increase. How could an increase in cell membrane permeability be assessed to back up the original explanation for ADC change?*

**Response**: ***We agree with the reviewer that a higher ADC value can be achieved by multiple underlying changes induced by treatment including cell death, diffusivity increase and increased permeability of cell membranes. A conventional ADC measurement, such as the method used in this study, cannot specifically tell which change is the dominant one. Hence we removed the sentence, and add this limitation of conventional ADC measurement in the discussion section.***

*R1.C13. Figure 7 does not show anything not shown in figure 8 so could be removed from the manuscript without losing any information.*

**Response: *Figure 7 was removed from the manuscript and figure 8 legend was updated per reviewer’s suggestion.***

***R1.C14.*** *Were the staining density maps for the immature vessels calculated independently from the overlaid fluorescence images or from a subtraction of COLIV from CD31? The density maps do not seem to correspond to the CD31 only staining in the fluorescence images. Please add scale bars to the histology images.*

**Response**: ***It was our oversight that we did not provide how the immature vessels were selected in this study. We added a paragraph in the method section to clarify this, along with new Figure 2 to describe the method more clearly. Briefly, it was based on the covariance of CD31 and COLIV images per patch in order to ensure that COLIV and CD31 were co-localized, not just highly expressed within the patch. Hence, it is not just a subtraction of COLIV staining density from CD31 staining density. The immature vessel map is defined as CD31 density masked by the covariance map. This is further explained by a dedicated paragraph with new Figure 2.***

***R1.C15.*** *The addition of an apoptotic marker would be beneficial to demonstrate whether 5FU is having the anticipated cytotoxic effect and H&E staining would more clearly demonstrate whether there is necrosis in the tumors than DAPI alone. When assessing response to a cytotoxic agent, cell death should be properly assessed. The addition of higher power snapshots of the histology would also add to the manuscript.*

**Response**: ***We agree with the reviewer that an apoptotic marker would provide useful information in addition to DAPI and Ki67 in this study. Since this is not possible to add a new marker to this study, we acknowledged this limitation in the discussion. We also agree with the reviewer that high power H&E images can be useful in assessing cell death. However, H&E staining was not included in this study as it is not straightforward to quantify an H&E image for a particular cellular property to match MRI measures. This was also noted as a limitation and potentially to be addressed in future studies.***

***R1.C16.*** *In the comparison between MRI and histology section please substitute ‘with tumor growth’ with ‘**in control tumors’. The lower cellular density in the MCT tumors cannot be described as a decrease unless compared to pre-treatment tumors, it should be described as being lower than in control tumors. The word ‘more’ is missing from the following sentence ‘the MCT tumor rim was also seen to have Ki76 staining than control tumors’, please also comment on the higher Ki67 staining in the core of MCT tumors.*

**Response**: ***Updated per reviewer’s suggestion***.

***R1.C17****. In figure 9C please use the same scales for ADC and Ki67 as in A&B.*

**Response**: ***Figure 9C has been revised to use the same scales for ADC and Ki67 as in previous 9A and 9B***. ***Since previous 9B showed only Ki67 and was redundant to 9C, it is now removed.***

***R1.C18.*** *Please clarify how you have come to the conclusion that the 5FU is having an anti-angiogenic effect in the rims of the MCT tumors if there are more immature vessels and higher DCE enhancement. As I understand it, the drug is likely to target the proliferating vascular endothelial cells that line immature vessels rather than the more mature vessels, so its likely that the more mature vessels would remain after treatment, and as you state in the conclusion, more mature vessels are less leaky.*

**Response**: ***We agree with the reviewer that our results can be interpreted in different ways, as they are somewhat different from what can be expected from a conventional anti-angiogenic treatment at a maximally tolerable dose.*** ***Our conclusion was based on the observation that, while MCT induced tumor volume reduction with increase in ADC, it still maintained more functional vasculature with contrast enhancement compared to the control group and pre-treatment level. This may suggest that the tumor vasculature was functionally normalized due to a weak, but still adequate amount of anti-angiogenic effect of MCT. However, we acknowledge that our study does not provide enough data to substantiate this and further studies are needed to investigate the anti-angiogenic effect of MCT. We clarified this in the discussion section.***

***R1.C19.*** *You state at the beginning of the discussion that you aimed to evaluate the cytotoxic and anti-angiogenic effects of 5FU but I am not convinced that you have appropriately validated the assessment of the cytotoxic effects. No assessment of cell death have been included in the manuscript*

**Response**: ***In this study, we have used ADC along with DAPI and Ki67 to assess the cell density and proliferation to assess the cytotoxic effect of 5FU. However, we agree with the reviewer that assessment of cell death using an apoptosis measure would have been a better marker. This is included as a limitation of this study.***

***R1.C20.*** *Please expand on why an area with high CD31 expression in the core of the tumor is likely to be becoming necrotic. You could assess the ECM to determine whether there is raised IFP in the core? This may also partially explain the lack of core enhancement if these regions are poorly perfused?*

**Response**: ***Thanks for the question. We agree with the reviewer that this observation of high CD31 expression in the core of the tumor with poor Gd enhancement is puzzling. We are not familiar with assessing the ECM to determine whether the IFP is raised in the core. As we described in the discussion, an increased IFP can be a factor contributing to poor perfusion. We also noted that the vessels in the core may be less permeable to Gd as most CD31-positive vessels in the core are also positive for COLIV. It is also interesting to note that some core areas have very low CD31 expression. It is not clear why these two contrasting areas exist in tumor cores. This requires further analysis in future studies. We clarified this in the revision.***

***R1.C21.*** *Please explain more fully how you concluded that there was no IVIM effect in these tumors, this is not explicitly mentioned in methods and analysis used monoexponential fitting.*

**Response**: ***Thanks for pointing out this oversight. We added more description in the method and the result sections regarding how we estimate ADC. Briefly, we used data with b>200 s/mm2 to minimize any influence of IVIM, although we did not see a strong IVIM effect as shown in Figure 7A. This has been clarified in the revised manuscript.***

**Reviewer 2.**

*The authors have investigated the use of diffusion MRI and dynamic contrast enhanced MRI (DCE-MRI) to evaluate response to chemotherapy in a breast cancer mouse model.
The used metronomic chemotherapy induces both anti-angiogenic and cytotoxic effects. The use of both DCE-MRI and diffusion MRI is therefore well motivated.
I do have some reservations with interpretation of the data.*

***R2.C1*** *Is the 3D-FLASH DCE-MRI sequence RF-spoiled ? Only with RF-spoiling the sequence is purely T1 weighted enabling accurate conversion to Gd concentrations possible.*

**Response: *Yes, our 3D-FLASH pulse sequence includes RF spoiling. It is clarified in the revised manuscript.***

***R2.C2.*** *I think it is quite unsatisfactory that no IVIM effect was observed particularly in view of the claim of changes in the vasculature due to the anti-angiogenic treatment.*

**Response: *We agree with the reviewer that it would be typically expected to see some changes in IVIM measures by anti-angiogenic treatment. That was why we also used 13 b-values with 6 of them less than 200 s/mm^2^. However, as shown in Figure 7A, we observed that the effect of IVIM was not clearly present in our diffusion decay curves, and led to difficulty in estimating IVIM parameters. Hence, we measured ADC using with b>200 s/mm^2^. It is puzzling why our data did not show IVIM effect clearly. This is why we also have a dedicated paragraph in the discussion on this issue. This has been clarified in the revised manuscript.***

***R2.C3*** *I don't understand the reasoning for the observed changes in the contrast enhancement in relation to (anti-) angiogenic effects. The contrast enhancement in the treated tumors decreases only slightly after treatment, to me suggesting minor changes in the tumor vasculature (at least you cannot tell from the MRI data). The chemotherapy clearly does not "cut off blood supply" as mentioned in the manuscript. For the control group a different mechanism may be at work as the tumor grows more rapidly and parts of the tumors may become less well perfusion which results in effectively lower contrast agent concentration at the later time point. In any case, the contrast enhancement does not only reflect blood supply, but also vessel permeability, which cannot be discriminated at this temporal resolution using a low-molecular contrast agent.*

**Response: *We agree with the reviewer that our results do not show vascular changes in a conventional anti-angiogenic therapy which is typically given at a maximal tolerable dose to maximize the anti-angiogenic effect as much as possible. Our interpretation of the data was that the low, but still effective anti-angiogenic effect of MCT prevented the treated tumor from having less contrast enhancement. We agree with the reviewer that the low contrast enhancement in the control tumors can be explained in multiple different ways including the increased IFP as mentioned in our discussion. As mentioned in the above response to Reviewer-1 (R1.C18), our conclusion was based on the observation that, while MCT induced tumor volume reduction with increase in ADC, it still maintained more functional vasculature with contrast enhancement compared to the control group and pre-treatment level. This may suggest that the tumor vasculature was functionally normalized due to the adequate amount of anti-angiogenic effect of MCT. However, we agree that our study does not provide enough data to substantiate this and further studies are needed to investigate the anti-angiogenic effect of MCT. We clarified this in the discussion section.***

***R2.C4.*** *Given the observations you make. How would you discriminate a successful treatment from an unsuccessful one based on DCE-MRI and diffusion MRI, if you wouldn't have had the control group ? The DCE-MRI curves pre- and post-treatment are essentially the same (within margins of error). The same holds for the ADC values. The tumors still grow. Based on these observations alone I would conclude that the treatment does not work. So why do you claim that diffusion MRI and DCE MRI are potential biomarkers to evaluate the treatment effect ?*

**Response: Thanks for raising this important question. Our assumption is that a successful treatment can be achieved by killing tumor cells without cutting off the blood supply throughout the treatment duration. We believe our data support the feasibility of using MCT for cytotoxic effect (as shown by reduction in tumor growth, increased ADC, and decreased DAPI and Ki67) whiling maintaining the functional vasculature possibly by a relatively weak anti-angiogenic effect (as shown by contrast enhancement in the core after treatment and higher CD31, COLIV and immature vessel densities). Since we need to measure both cellular and vascular changes, we believe our data also support the use of both diffusion MRI and DCE-MRI. However, we also acknowledge that this study is limited in many ways as mentioned in the discussion. Further studies need to be conducted to establish a robust way of using both MRI methods together to evaluate the cytotoxic and anti-angiogenic treatment effects. This has been further clarified in the revised manuscript.**
